# Public Health Insurance Status and Utilization of Healthcare Services Across India: A Narrative Review

**DOI:** 10.7759/cureus.54308

**Published:** 2024-02-16

**Authors:** Vaibhavi Shende, Vasant Wagh

**Affiliations:** 1 School of Epidemiology and Public Health, Datta Meghe Institute of Higher Education and Research, Wardha, IND; 2 Community Medicine, Datta Meghe Institute of Higher Education and Research, Wardha, IND

**Keywords:** health insurance knowledge, utilization of health care, health insurance scheme, health insurance literacy, health insurance

## Abstract

Health insurance literacy gauges how knowledgeable people are regarding the comparison of health insurance plans to find out the optimal health plan that suits their needs and preferences. Enrolling in a comprehensive plan and proactively addressing health and financial aspects can fortify the stability of families. The plan needs to be used effectively by adapting to evolving circumstances and prioritizing the well-being and prosperity of the household. Having public health insurance can significantly impact an individual's utilization of healthcare services. Having health insurance encourages individuals to promptly seek medical attention without hesitating or avoiding treatment due to financial worries. This results in higher utilization of healthcare services, encompassing routine check-ups, preventive care, and timely intervention for illnesses and injuries. Public health insurance can also improve access to specialized care and expensive treatments that may otherwise be unaffordable for individuals without insurance. By having health insurance, individuals and families can experience a decrease in the economic strain associated with healthcare expenses, thereby enhancing the accessibility and affordability of healthcare services.

## Introduction and background

Public health insurance status refers to whether or not an individual is covered by a government-funded health insurance program. The application of health insurance principles and the understanding of health insurance terminology are included in health insurance literacy (HIL). The broad definition of health services utilization is the consumption of and accessibility to services for the aim of increasing health and well-being, preventing and treating health problems, or getting health-related information [1]. With separate public and private healthcare systems, India has a complicated and heterogeneous healthcare system. In India, health policies are shaped by a commitment to equity, reflected in the substantial disparity between government expenditures on public and private healthcare, giving priority to the needs of the impoverished and disadvantaged [2]. This category of insurance facilitates entry to a range of healthcare services, including consultations with doctors, hospital accommodations, prescription medications, and preventive screenings. The status of the utilization of healthcare services is significantly influenced by the pivotal role played by public health insurance. It guarantees individuals access to essential medical care, fostering improved health outcomes, and overall well-being.

In the 1970s, the term "health literacy" pertained to an individual's capacity to navigate the myriad demands involved in promoting and maintaining health within today's society. Limited health literacy is linked to difficulties in understanding health information, a restricted grasp of illnesses, and decreased adherence to medication. As a result, it plays a role in contributing to overall poor health, heightened risk of mortality, suboptimal utilization of healthcare resources, escalated costs, and the perpetuation of health disparities [3]. The financing of healthcare has undergone continuous change in many nations, particularly in developing nations. As evidenced by the Sustainable Development Goals (SDGs), Universal Health Coverage and Global Health movements, and other international development agendas, healthcare financing mechanisms at the national level have transcended the realm of exclusive national-level policy discourse and considerations [4]. After life and vehicle insurance, health insurance is the fastest-growing insurance market in India. Several significant factors including the growing middle class, rising hospitalization costs, digitization, and rising awareness have contributed to the expansion of the health coverage market in India. Health insurance providers must innovate their offerings as well as their distribution networks and include them as a safety net that judiciously addresses and creates an insurance program that meets the needs of the clients [5].

 In low- and middle-income countries (LMICs), community-based health insurance (CBHI) has come about as an alternative to out-of-pocket (OOP) medical expenses. There is significant variance in the impacts and coverage attained, even though CBHI schemes may have a considerable potential to improve financial security and enhance utilization among their enrolled populations [6]. Irrespective of color, religion, political affiliation, economic status, or social standing, every individual possesses the inherent right to access the highest achievable standard of health. The importance of literacy in healthcare is paramount [7]. Health insurance emerges as a complex and costly product that individuals obtain and use throughout their lifetimes. The establishment of federal and state health insurance marketplaces is designed to improve access to insurance for the uninsured and simplify the process of selecting and purchasing health insurance from the private market [8]. The majority of healthcare professionals participating in a study by Mosadeghrad in Tehran emphasized that a shortage of resources substantially hampers the quality of healthcare services [9]. Most healthcare expenses in developing nations are covered by patients themselves through OOP payments rendered at the moment and place of receiving treatment. In India, private investors cover 70% of healthcare costs, of which 86% come from OOP expenses [10]. Beneficiaries should be thus able to be conveniently treated for a wide range of medical disorders at the price and level of care they choose [11].

## Review

**Figure 1:**
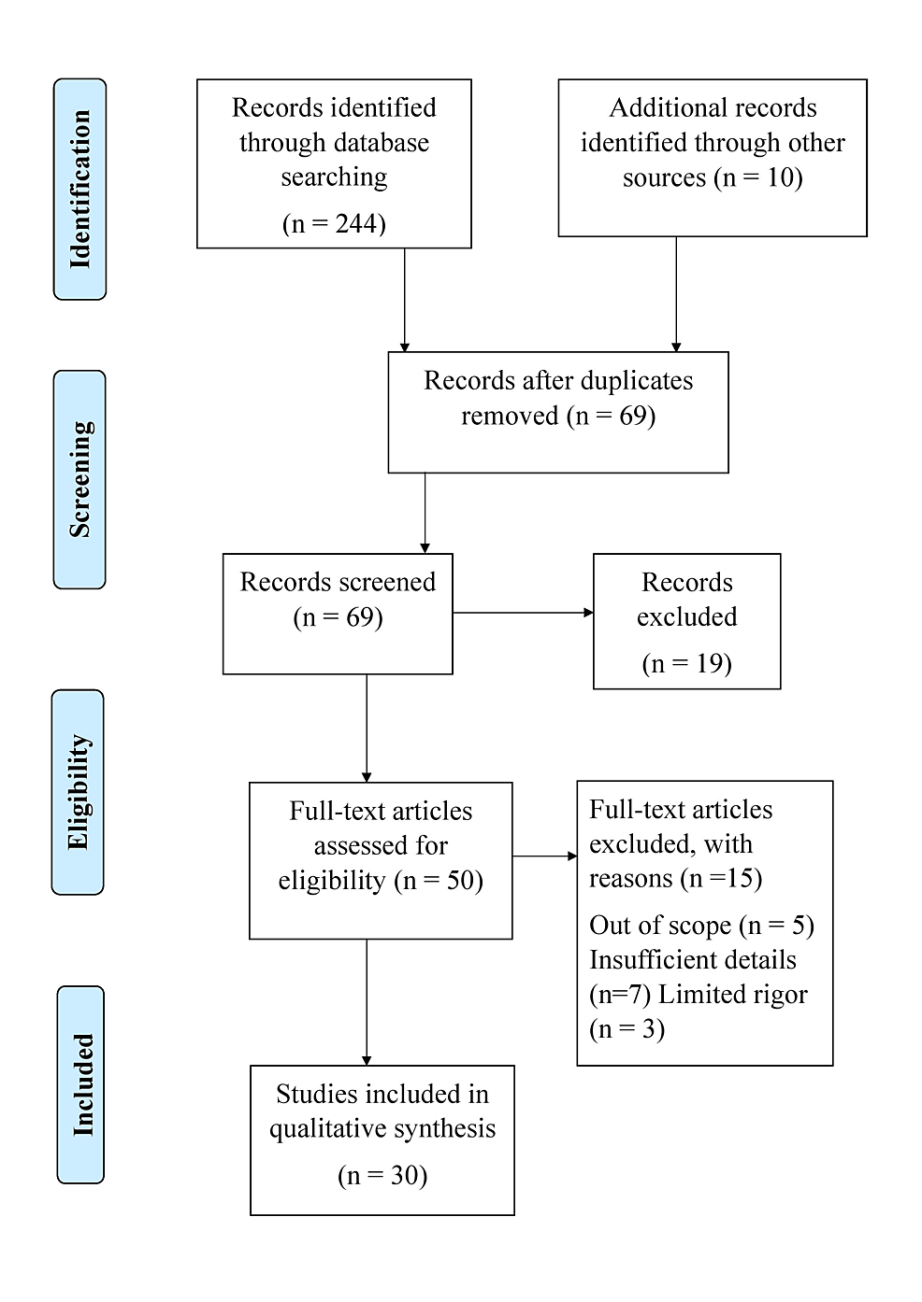
Study selection process for this review

PubMed, Google Scholar, and WHO databases were searched with the following search terms: ((public health insurance) AND (utilization of health care)) AND (in India). Inclusion criteria included the availability of free full text, articles published in the English language, and from 2014 to 2024. Of a total of 50 studies finally assessed for eligibility, 30 were analyzed in this review. The article selection process is shown in Figure 1. 

Level of health literacy in India

A vital component of patient care is health literacy. Health literacy encompasses an individual's ability to acquire, communicate, process, and comprehend vital health-related information and services. This proficiency empowers individuals to make informed decisions regarding medical matters. It appears that residents with a lower HIL have a harder time locating a good health insurance provider. This highlights the need to research the idea of health insurance coverage in other countries where people are expected to actively participate in selecting a health insurance plan [12]. As per the study by the Ministry of Statistics and Programme Execution, Government of India, India's literacy rate for the 2020-21 fiscal year is 77.7% [13]. Health literacy is substantially connected with health outcomes, health behaviors, and social determinants of health. It has numerous implications for medical treatment, health education, and health promotion. As a result, health literacy is seen as one of the most significant problems in public health and healthcare and is being addressed more and more in current healthcare and social policies [14].

Over the last five years, since 2007, India has witnessed the initiation of diverse programs, with numerous state governments and the federal government actively participating in the adoption of health insurance initiatives. The Indian government's pledge to increase public healthcare spending is one of the drivers for the launch of these kinds of initiatives [5].

Health insurance schemes: (mis)trust and misunderstanding

The population-based insurance notion of risk pooling appears to be poorly understood. A study conducted by Kusuma et al. revealed that only 18% of the urban poor currently have access to health insurance, leaving a staggering 82% without coverage [15]. Additionally, a mere 9.4% of eligible households are enrolled in health insurance programs, highlighting the significant gap in coverage within this population. Participants of the study said they would rather pay OOP for treatments when needed and do have not much trust in health insurance plans. They used anecdotes regarding bad experiences friends or relatives had with different kinds of insurance as justification. The extent to which people have faith in government-backed insurance programs is related to the broader issue of consumer confidence in the government and the extent to which these government agencies are seen fulfilling their obligation to protect the citizens.

An overview of India's current insurance programs

**Figure 2:**
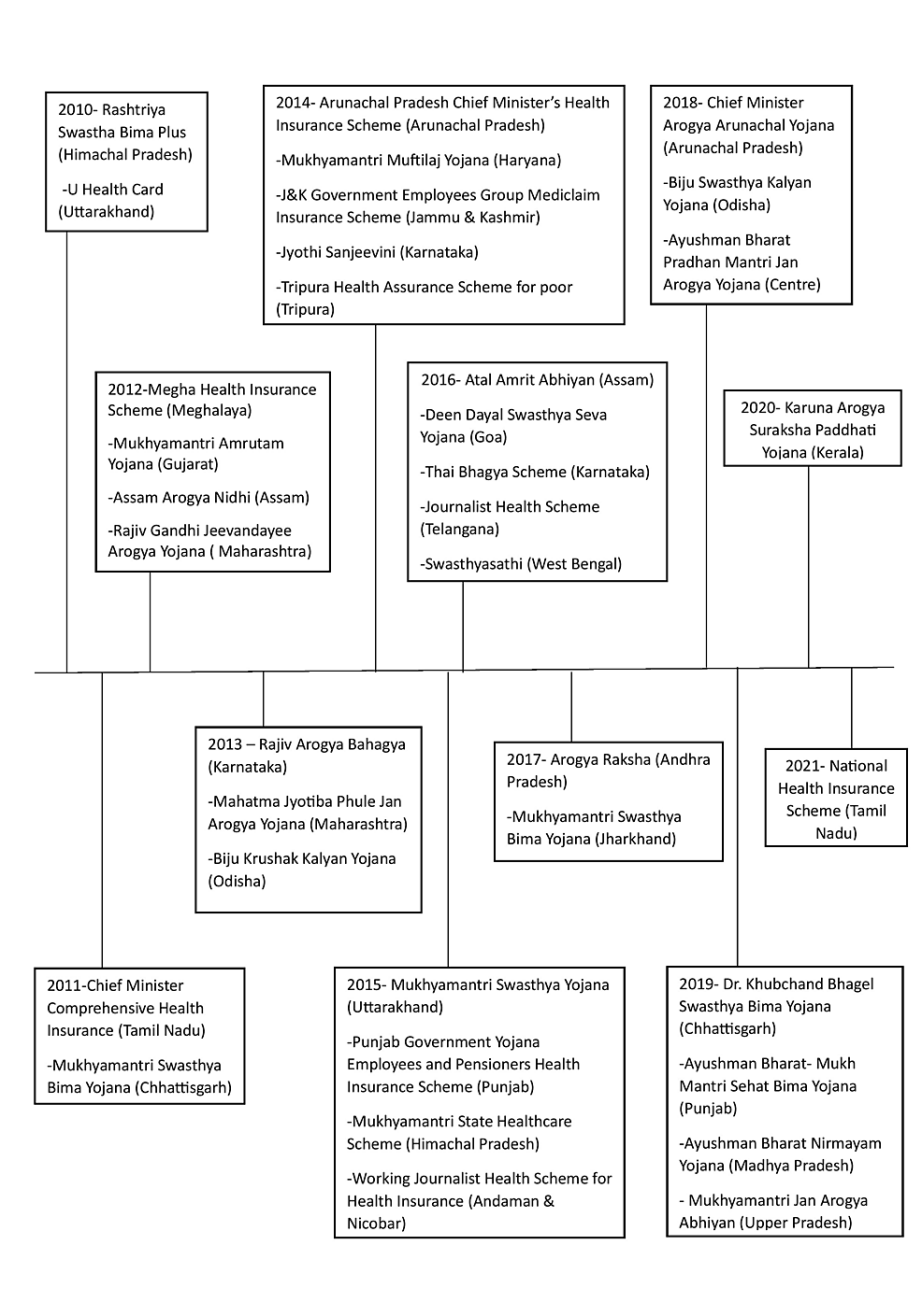
Timeline of health insurance programs funded by the government in India and social health insurance programs (2010–2022).

To safeguard families that fall below the federal poverty line (BPL), the Indian government introduced Universal Health Insurance (UHI) in 2003. This program partially subsidizes insurance premiums. It has been proposed in the fields of health care and health policymaking that health insurance, by lowering emergency healthcare costs for all socioeconomic levels, might serve as a vital safety net for low- to middle-income nations residents. However, only a tiny portion of Indians had health insurance until recently. Only around 10% of public employees in India have coverage by the Central Government Health Scheme (CGHS) created in 1954 and the Employees' State Insurance Scheme (ESIS) introduced in 1948. Various factors contribute to building health policy or we can say some factors have drawn Indian policymakers' attention to health insurance like the high cost of illness, minimal public healthcare spending, high private healthcare costs, and restricted coverage by the current health insurance programs. In India, only 1.04% of the total gross domestic product (GDP) is allocated to public health spending. The estimated overall health spending, including the contribution from the private sector, is 4% of the GDP. Approximately 30% of the overall expenditure comes from the public sector [16]. The goal of bringing health insurance to low-income individuals living in informal economy areas necessitates the customization of benefit packages and premium amounts to local circumstances [17]. It is important to remember that the wave of healthcare privatization in the 1990s was followed by the establishment of multiple state-level government-funded health insurance schemes (GFHIS). Inadequacy in the public healthcare system increased during this time due to a decrease in government expenditure in the healthcare industry. Later, the Centre and several states adopted this concept, as shown in Figure 2 [11].

Health insurance and utilization of healthcare services

Most studies demonstrated that lower HIL was linked to greater evasion of an extensive array of healthcare services or lower healthcare utilization. Several studies also showed that low HIL was linked to insufficient use of certain significant services such as follow-up with prescription medication schedules for chronic conditions and other preventive medical visits, as well as primary care visits [18]. Also, there are various benefits to vulnerable groups like maternal and child health. In LMICs, encouraging women to give birth in healthcare facilities has been a pivotal global strategy aimed at reducing maternal and perinatal fatalities [19]. However, low-income residents of rural regions are not benefiting from the system at all because of its numerous failures [20].

A study by Mohapatro et al. shows that people with health insurance, whether it be private or public, are less likely to have positive OOP expenses than those without insurance. It also indicates that the government pays twice as much for healthcare for the poorest citizens. According to the study, government-funded insurance programs showed a coverage success rate of 15%, whereas public-funded insurance plans had a higher coverage rate of 12.8% [21].

In a study by Edward et al. in the United States, more than half (51%) and approximately half (48%) of the sample of 15,168 people had insufficient HIL with regard to understanding concepts about health insurance and confidence in using insurance, respectively [22]. The benefits of health insurance should be adjusted based on the varying medical needs of individuals [23]. The idea that healthy individuals don't require health insurance is among the most widespread deterrents to its purchase. The gravity only becomes apparent when a medical emergency arises [13]. Health insurance is one of the most important tactics that can support the advancement of universal healthcare coverage through increased usage of healthcare services and financial protection [24].

Health insurance coverage in different states of India

India's health insurance situation is far from ideal. based on information obtained from the National Family Health Survey report for India [5]. About one-sixth (16%) of people with insurance are covered by the Rashtriya Swasthya Bima Yojana (RSBY), and nearly half (46%) are insured by a state health insurance scheme. The CGHS and the Employee State Insurance Scheme (ESIS) cover about 4-7% of men and 3-6% of women, respectively. The Andaman & Nicobar Islands and Jammu & Kashmir have the lowest coverage (less than 15%), while Rajasthan and Andhra Pradesh have the largest percentage of families covered by health insurance or finance schemes (88% and 80%, respectively). Data from the National Family Health Survey India report indicates that one family member has regular health insurance coverage in 41% of homes and between 2019 and 2021, just 30% of women and 33% of males between the ages of 15 and 49 were protected by insurance for health or finance programs. The lack of awareness, limited knowledge, and economic constraints are the primary factors impeding health consciousness. Furthermore, the public healthcare system is facing significant funding deficits, which is hindering its capacity to adequately address the healthcare needs of the population [25]. The promise of affordable and accessible healthcare is threatened by inadequate infrastructure and a workforce devoid of training. Fascinatingly, instances of fraud are documented in which the insured attempts to collect payments for hospital stays that were never incurred [26]. Table 1 depicts the percentage of household persons who are insured for healthcare services in various states of India [25].

**Table 1:** Distribution of the percentage of households across states that have at least one member with health insurance.

States	Percentage of households with at least one person who is insured for healthcare
Bihar	17%
Jammu & Kashmir	14%
Tripura	36%
West Bengal	34%
Karnataka	32%
Assam	67%
Telangana	69%
Mizoram	50%
Gujarat	44%
Himachal Pradesh	39%
Kerala	58%
Nagaland	22%
Delhi	25%
Sikkim	28%
Maharashtra	22%
Chhattisgarh	71%
Goa	73%
Rajasthan	88%
Andhra Pradesh	80%

The findings from Tipirneni et al.'s study suggest that a diminished health insurance literacy level could be associated with an increased tendency to forgo both preventive and non-preventive care. Regardless of an individual's proficiency in health insurance literacy, it seems essential that health insurance principles be communicated in an understandable way to lawmakers, health plans, and healthcare providers. This approach is crucial to promote the appropriate utilization of specific healthcare services, particularly those related to preventive health measures [27]. According to health and family welfare statistics in 2022, the breakdown of a person by health expenditure coverage is shown in Table 2 [28].

**Table 2:** Individual breakdown based on health spending coverage. PSU: public sector undertaking

Sector	Percentage of people who aren’t covered		Percentage of those protected by PSU/Government as an Employer				
		Government-sponsored insurance program	PSU/Government as an Employer	Employer-sponsored health insurance (apart from PSU/government)	Coordinated by the home with the insurance provider	Others	All
Rural	85.9	12.9	0.6	0.3	0.2	0.1	100.0
Urban	80.9	8.9	3.3	2.9	3.8	0.2	100.0

The existing coverage is lower than anticipated due to overlaps among different health insurance plans and the reality that not every household eligible for government insurance subsidies is receiving coverage at this time. However given that government-subsidized programs are anticipated to gradually cover those who are eligible, India has a 70% prospective coverage rate for health insurance based on the current situation [29]. Information facilitators are becoming more widely acknowledged as sources that customers use to find out about health insurance [30].

## Conclusions

Because it covers a larger portion of the population financially, public health insurance can positively impact the utilization of medical services. This frequently leads to better overall health outcomes by increasing access to early detection, timely treatment, and preventive care. To maximize their influence on healthcare utilization, public health insurance programs must be implemented and managed well. It is important to remember, that HIL might differ between geographical areas and demographic groupings. Human public health insurance knowledge varies greatly. Health insurance knowledge can be influenced by a variety of factors, including access to information, socioeconomic situation, and level of education. To support consumers in making educated choices about their coverage and successfully navigating the healthcare system, efforts to increase health insurance literacy frequently concentrate on offering clear, easily available information. public health coverage. HIL includes awareness of available plans, coverage details, eligibility criteria, and how to navigate and utilize the services effectively. Improving public health insurance literacy is crucial for enabling people to make knowledgeable healthcare decisions and maximize the benefits provided by public insurance programs.

Health insurance in India enables individuals to take the benefits of the medical services covered by their insurance policies without bearing the full financial burden themselves. Policyholders can seek medical treatment at network hospitals, and expenses are typically settled directly between the insurer and the hospital. Individuals must be aware of their policy coverage, including inclusions, exclusions, and claim procedures, to maximize the benefits of health insurance. Under the policy terms, individuals can file claims for regular health check-ups, hospitalization, and other qualified medical expenses.
